# The crowding-out effect of physical fitness activities on medical expenditure in the aged group

**DOI:** 10.3389/fpubh.2024.1425601

**Published:** 2024-11-15

**Authors:** Tao Liu, Yujiao Yao, Zhandong Yang, Kaigeng Li, Tao Yu, Yalong Xia

**Affiliations:** ^1^Chengdu Shude High School, Chengdu, Sichuan Province, China; ^2^School of International Studies, Sichuan University, Chengdu, China; ^3^School of Leisure Sports and Tourism, Beijing Sport University, Beijing, China; ^4^School of Media and Law, NingboTech University, Ningbo, China; ^5^School of Business, University of Chinese Academy of Social Sciences, Beijing, China

**Keywords:** physical activity, healthy aging, medical expenditure, crowding-out effect, tobit model

## Abstract

**Introduction:**

China is facing the challenge of “deep aging”, and promoting healthy aging has become a key research topic. Both medical care and physical exercise are important for health, but while medical services focus on treating illness, physical fitness activities focus on prevention, making them a more effective approach for promoting healthy aging.

**Methods:**

This study uses data from the China Health and Retirement Longitudinal Study (CHARLS), focusing on individuals aged 60 to 80, to investigate the relationship between physical fitness activities and medical expenditures. A Tobit model was employed to analyze the data.

**Results:**

(1) Active participation in physical fitness activities significantly improves the health of older adults, making physical activity an essential pathway to achieve healthy aging. (2) Participation in sports and fitness activities leads to a crowding-out effect on medical expenditures, significantly reducing healthcare costs for participants. (3) High-intensity physical activities are most suitable for individuals aged 60-65, moderate-intensity activities for those aged 66-70, and low-intensity activities for those aged 71–80.

**Discussion:**

(1) Policies should focus on raising awareness of physical fitness benefits among older adults, encouraging regular physical activity to improve health and reduce medical costs. (2) A shift from treatment to prevention in health management is needed, promoting exercise as a cost-effective way to reduce healthcare spending. (3) Age-specific fitness guidelines should be developed to provide tailored exercise recommendations for different older aldult age groups.

## 1 Introduction

As of the end of 2023, the population of persons over 65 and above in China reached 297 million, accounting for 21.1% of the total population. It is projected that by around 2035, this demographic will exceed 400 million, surpassing 30% of the total population, which will position China in a state of deep aging. The promotion of healthy aging in China has thus become a focal point of recent research. China has implemented the Healthy China Strategy, which aims to improve the health of the entire population. The Outline of the “Healthy China 2030” Plan explicitly proposes that the number of people who regularly participate in physical exercise should reach 530 million by 2023. It emphasizes the concept of front-end health services, promoting proactive health measures over reactive medical interventions (1). It is clearly proposed that the number of people who regularly participate in physical exercise should reach 530 million by 2023. The Healthy China Initiative (2019–2030) proposes a national fitness initiative. Lack of physical activity has become one of the main causes of chronic diseases. Medical care and sports are both key pathways to improving health. However, the health benefits derived from medical services are in the nature of post-event health, whereas the health benefits from physical fitness activities represent proactive health. Consequently, the preventive aspect of physical fitness activities is gaining increasing attention. The World Health Organization reports that 60% of health determinants depend on personal lifestyle, of which physical fitness activities are a component. Therefore, to comprehensively elevate the health level of the older adult, physical fitness activities should be included in the government’s considerations, just like medical services.

In fact, as early as 2006, Suzhou began exploring the integration of sports with medical care, allowing a portion of the basic medical insurance personal account funds to be used for fitness expenses, with over 30,000 individuals having spent their medical insurance funds on physical fitness activities (2). Currently, not only have eight prefecture-level cities in Jiangsu Province, including Suzhou, Nanjing, and Changzhou, implemented the policy of “using medical insurance cards for fitness,” but other cities nationwide like Shenzhen and Chongqing have also launched similar policies (3, 4), aiming to reduce the medical burden on the older adult through proactive health management. Whether the investment in physical fitness activities can effectively reduce medical expenses while maintaining and enhancing health levels, i.e., whether physical fitness activities have a crowding-out effect on medical expenditure, is a crucial piece of evidence supporting the rational integration of medical insurance cards with fitness activities. Despite extensive and detailed theoretical discussions by many scholars on the aforementioned issues, there is still a lack of concrete evidence and empirical verification regarding the negative correlation between physical fitness activities and medical expenses in China. The research into whether physical fitness activities can crowd out medical expenses for the older adult is precisely what provides objective evidence and a realistic basis for policies integrating physical fitness with medical care.

Physical fitness activities can indeed improve the health levels of the older adult. The World Health Organization (WHO) has identified a lack of physical exercise as the fourth leading cause of global mortality. Preliminary research confirms that regular physical fitness activities can improve health levels. Physical activity not only helps to improve the physical health of older adults (5), but also helps to enhance their mental health (6). Appropriate physical exercise can effectively prevent chronic non-communicable diseases and also reduce mortality rates (7, 8). Lee and others have conducted research on the prevention of diseases and health promotion through physical fitness activities, confirming that such activities can prevent diseases such as coronary heart disease, hypertension, stroke, and depression, as well as promote health levels, including functional health and cognitive functions (9, 10). Individuals over 60 in the Tsinghua University community and found that physical activity could effectively reduce the risk of chronic diseases. It is evident that physical fitness activities are a crucial factor in improving health levels (11).

Further detailed research on physical fitness activities and health shows that aerobic exercises can slow cognitive decline in the persons aged 65–74 (12). Additionally, aerobic exercises can effectively enhance the executive functions of the older adult (13, 14). Taijiquan and qigong are traditional Chinese therapeutic disciplines with several health benefits (15). Taijiquan has been found to effectively improve the physical health levels of the older adult (16–18). Although participating in physical fitness activities is a personal choice, it reflects the interaction within groups (19, 20). As a personal choice, actively participating in physical fitness activities can strengthen the body, release stress and negative emotions. As a social method, it can enhance communication among the older adult, reducing feelings of loneliness. Thus, physical fitness activities can be a significant pathway to achieving “healthy aging” (21).

During the “Thirteenth Five-Year Plan” period, population aging has brought greater pressure to health services for the older adult. Lack of physical activity can lead to higher health care costs (22). The number of older and disabled older adult people has continuously increased during the aging process, and this group has a higher frequency of utilizing medical services, placing significant pressure on individuals, families, and the healthcare system. Whether physical fitness activities can reduce medical expenses for the older adult has significant practical importance.

Compared the outpatient expenses of urban residents and found that those over 65 years of age spent more than the general population (23). Physical fitness activities could be a new breakthrough in addressing the medical burden brought about by aging. Research by Ackermann et al. indicates that regular participation in community physical fitness activities can reduce medical costs (24). The implementation of a national fitness plan can improve the health level of the older adult and thereby reduce their medical expenses (25). Munro et al. provided $854,700 for physical fitness activities twice a week for over 10,000 volunteers aged 65 and over in the United States (26). The result was an estimated savings of about $601,000 in hospital medical expenses, meaning for every dollar invested in bi-weekly physical fitness activities, the weekly hospital medical costs could save $0.7107. Research by Pratt et al. with American seniors as subjects yielded similar conclusions, showing that seniors who regularly engage in physical fitness activities save an average of $1,019 per year on medical expenses compared to those who do not (27). Focusing on the medical expenses for hip fractures, if everyone were to engage in regular physical activity or exercise according to the ACSM standards, the corresponding medical costs could be reduced by 50% (28). Ding’s empirical analysis of data from 142 countries in 2013 showed that insufficient physical activity among residents of these countries leads to an average of $53.8 billion in additional medical costs each year (29). The inverse relationship between physical activity and the cost of chronic disease was found using Brazil as the study population. Regular exercise had an advantage over medication as a treatment option for chronic disease, with a cost-utility ratio of $3.21/quality-adjusted life year (QALY) for regular exercise compared with $3.92/QALY for the medication-only group (30).

In summary, physical fitness activities not only improve the health levels of the older adult but also alleviate their medical expenditure burden (31). This paper’s research objectives and expected theoretical contributions and innovations are reflected in the following aspects: First, it examines the “crowding-out effect” of physical fitness activities on medical expenses, providing reliable evidence to support the policy concept of “integrating sports with medical care.” The impact of physical fitness activities on health should not only be considered theoretically but also empirically verified. The author verifies that whether physical fitness activities and their intensity can reduce the medical expenses of the older adult, that is, the existence of the “crowding-out effect” of physical fitness activities on medical expenses. If the “crowding-out effect” is proven to exist, it will not only provide a rational basis for the new development model of integrating sports with medical care but also offer a practical reference for the realization of “healthy aging” in China. Second, it refines the measurement of the intensity and duration of physical fitness activities and their impact on the health of the older adult. Both theoretical and quantitative analyses have confirmed that participating in physical fitness activities can effectively improve the health levels of the older adult. However, the intensity and duration of participation in physical fitness activities—two key factors—are worth further in-depth study to determine their impact and extent of influence on the health levels of the older adult. The analysis results can provide references for the design of physical fitness activity programs for the older adult. Third, it constructs a more comprehensive health composite index to measure health levels. Current research on the impact of sports on health mostly focuses on the improvement of physical functions and often overlooks the influence of sports on mental health. By constructing a frailty index, this paper builds a comprehensive health index from both physical function and mental health perspectives to more comprehensively analyze the impact of physical fitness activities on the health of the older adult.

## 2 Materials and methods

### 2.1 Source of data

This paper utilizes 2015 data from the China Health and Retirement Longitudinal Study (CHARLS), a large-scale interdisciplinary survey project jointly implemented by Wuhan University and Peking University. The CHARLS aims to collect a set of high-quality micro-data representing households and individuals of middle-aged and older people aged 45 and above in China to analyze China’s aging population. The CHARLS covers 150 county-level units, 450 village-level units, and about 10,000 households in China. The data will be rolled out to the academic community 1 year after the survey is completed. The CHARLS questionnaire includes basic information about the individual, family structure and financial support, health status, physical measurements, health care utilization and health insurance, work, retirement and pensions, income, consumption, assets, and basic community information. Considering that the fitness indicators for the persons aged above 80 primarily reflect in medical and physical fitness aspects, the research subjects were set between the ages of 60 and 80. Regarding the sample size, measurement errors from the questionnaire are unavoidable, and the extreme values of the top 1% and bottom 1% of the income-related variables in 2015 were excluded to ensure the robustness of the data. Meanwhile, considering the problem of omitted variables in the model, the 2011 tracking survey data were matched with the 2015 data using the identity codes corresponding to the individuals. Furthermore, data regarding physical fitness activities were randomly surveyed for only half of the entire sample size. After screening, the final sample size for this study was determined to be 1,877.

### 2.2 Construction of the model

Building on Grossman’s health production function (31), this article views physical fitness activities as an investment element in health and incorporates them into the health function. Thus, the input–output model for physical fitness activities on health is:


(1) $$\mathrm{Healt}h_{i}=\alpha_{o}+\alpha_{1}\mathrm{Spor}t_{i}+\sum \alpha_{j}X_{ji}+\varepsilon_{i}$$

In Equation 1, $\mathrm{Healt}h_{i}$ denotes the health level of the older adult, $\mathrm{Spor}t_{i}$ represents physical fitness activities, $X_{ji}$ stands for control variables, $\alpha_{1}$ indicates the marginal output of activity input on health, and $\varepsilon_{i}$ is an unobservable random variable. The physical fitness activities variable in Equation 1 encompasses three layers of meaning: whether to participate in physical fitness activities, and during such participation, the intensity and duration of the exercise.

To further validate whether participation in physical fitness activities can alleviate the medical burden of the older adult, medical expenditure (Exp) is taken as the dependent variable. Some older adult have zero medical expenses, which presents a “left-censored” phenomenon. Therefore, the Tobit model is employed to investigate the crowding-out effect of physical fitness activities on the medical expenses of the older adult, and its specific form is:


(2) $$\exp_{i}^{*}=\beta_{0}+\beta_{1}\mathrm{Spor}t_{i}+\beta_{2}\mathrm{Healt}h_{i}+\sum \beta_{k}W_{ki}+e_{i}$$

(3) $$E_{i}=\{\begin{array}{l} E_{i}^{*},\;\mathrm{if}\;E_{i}^{*}>0\\ 0,\;\mathrm{if}\;E_{i}^{*}=0 \end{array}$$

In Equation 2, $\exp_{i}^{*}$ denotes the latent variable for medical expenses, the explanatory variables include control variables (W), and introduce a health level variable, with the meaning of physical fitness activities being the same as in Equation 1. Equation 3 focuses on the marginal effect (ME) of sports health activities on medical expenses, that is, $\mathrm{ME}=E\left(\exp\left|\mathrm{Sport},\;\mathrm{Health},\;W,\right.\;\exp>0\right)$ . $\beta_{1}$ is used to depict whether there is a crowding-out effect of physical fitness activities on medical expenditures; if $\beta_{1}$ passes statistical testing and its effect is negative, it is believed that participation in physical fitness activities reduces the medical expenses of the older adult, i.e., the crowding-out effect exists. In this paper, the following regression analysis is performed using Stata version 17.0.

### 2.3 Selection and processing of variables

#### 2.3.1 Overall health level

This paper utilizes the construction method of frailty index to construct the comprehensive index that can measure health - overall health (32, 33). It contains both physical functioning and mental health dimensions. The CHARLS database about physical functioning dimension includes 26 aspects and mental health dimension includes 12 aspects. With the specific calculation formula being:


(4) $$\mathrm{Healt}h_{i}=1-\frac{1}{n_{1}+n_{2}}\left(n_{1}\cdot \overset{n_{1}}{\underset{j=1}{\sum }}q_{j}+n_{2}\cdot \overset{n_{2}}{\underset{r=1}{\sum }}p_{r}\right)$$

In Equation 4, *Health_i_* is the frail health index, which represents the overall health level of the older adult, belongs to the continuous variable, The value of *Health*_*i*_ ranges from 0 to 1, the larger the value indicates that the *i*th individual overall health level is better. *q_j_* denotes the score corresponding to the *j*th question in the physical function dimension; *p_r_* represents the score for the *r*th question in the mental health dimension; *n* indicates the number of questions *n_1_* = 26, *n_2_* = 12.

#### 2.3.2 Medical expenses

The variable for medical expenses is selected to measure the medical burden of the older adult, meaning the greater the amount of medical expenses, the heavier the medical burden. The CHARLS database includes costs associated with self-treatment, outpatient services, hospitalization, and corresponding transportation expenses each month, with the final medical expense variable being the sum of all direct and indirect costs. To standardize the data and mitigate heteroscedasticity, medical expenses are converted to monthly figures, with the unit set to “ten thousand yuan/month.”

#### 2.3.3 Intensity and duration of physical fitness activities

According to the standards of the National Fitness Guidelines (SGAS, 2017) set by the General Administration of Sports, CHARLS questionnaire classifies physical fitness activities into “high intensity,” “medium intensity,” and “low intensity”: High-intensity exercises are those with a strong physical stimulus, such as running, fast cycling, and high-paced aerobics, where the heart rate exceeds 140 beats/min; medium-intensity exercises provide a moderate stimulus, with a heart rate typically between 100 and 140 beats/min, such as brisk walking, jogging, and Tai Chi; low-intensity exercises have a minimal physical impact, with a heart rate usually not exceeding 100 beats/min, like walking. The variable for the duration of physical fitness activities is weekly exercise time, which is calculated by multiplying the mid-value of each group in the unequal interval series for daily exercise time by the number of exercise days per week to obtain weekly exercise duration.

#### 2.3.4 Control variables

The Blue Book on the Development of Healthy Aging in China - Research and Policy on Positive Response to Population Aging (2023–2024) clearly indicates that the three major factors affecting the health of the older adult in China are economic and social factors, lifestyle and medical services. Based on current research results (18, 30, 34) and China’s unique national conditions, this paper divides the control variables into four areas (see Table 1): Socio-demographic characteristics include: marital status, age at marriage, sex, age, household registration, and educational attainment (expressed as years of schooling). Economic factors include: whether or not receiving pension, whether or not enrolled in health insurance, and logarithm of disposable income. Lifestyle includes: quality of sleep, whether or not one smoke and whether or not one drinks alcohol; Environmental factors include: whether or not the toilet can be flushed (1 = yes) and whether or not there is running water; and the health endowment consists of: chronic diseases (replaced by the percentage of types of chronic diseases suffered, where the types of chronic diseases are 16) and the variable of physical conditions prior to age 15.

**Table 1:** Results of descriptive statistical analysis of core variables.

Age group (years)	Physical fitness activity intensity (Times/min)	I: Mean comparison						II: Participation rate (%)	III: Average exercise duration (Minutes/week)
		Overall health			Medical expenditure (Unit: 10,000 RMB/month)				
		Participate	Do not participate	Difference 1	Participate	Do not participate	Difference 2		
Total	High Intensity	0.8478	0.8322	0.0156	0.2015	0.2584	−0.0569	35.22	21.8619
	Moderate Intensity	0.8493	0.8179	0.0314	0.2317	0.2446	−0.0129	55.93	18.3325
	Low Intensity	0.8433	0.7971	0.0462	0.2248	0.2377	−0.0129	79.19	15.0623
60–65	High Intensity	0.8594	0.8465	0.0129	0.2224	0.3366	−0.1142	47.71	22.8944
	Moderate Intensity	0.8575	0.8386	0.0189	0.2574	0.3529	−0.0955	64.12	19.0049
	Low Intensity	0.8536	0.8436	0.0100	0.2461	0.2860	−0.0399	80.32	15.3902
66–70	High Intensity	0.8466	0.8406	0.0060	0.2489	0.2518	−0.0029	47.15	22.1439
	Moderate Intensity	0.8462	0.8360	0.0102	0.2049	0.2675	−0.0626	62.81	19.8365
	Low Intensity	0.8476	0.7983	0.0493	0.2111	0.2541	−0.0430	81.63	16.4162
71–75	High Intensity	0.8356	0.8313	0.0043	0.2254	0.2913	−0.0659	38.31	21.6806
	Moderate Intensity	0.8443	0.8138	0.0305	0.1975	0.2862	−0.0887	55.92	18.2027
	Low Intensity	0.8353	0.7831	0.0522	0.2011	0.2512	−0.0501	78.73	15.8526
76–80	High Intensity	0.8400	0.8371	0.0029	0.1072	0.1752	−0.0680	17.61	17.5275
	Moderate Intensity	0.8429	0.7980	0.0449	0.1334	0.1842	−0.0508	41.45	13.6501
	Low Intensity	0.8301	0.7415	0.0886	0.1528	0.2208	−0.0680	74.74	12.0989

In summary, Table 2 present the description statistics for the control variables examined in this study.

**Table 2:** Results of descriptive statistical analysis of control variables (*N* = 1877).

Variable name	Mean	Standard deviation
Age	68.01	5.75
Educational attainment	5.31	4.25
Income (Ten Thousands Yuan)	2.59	4.11
Physical conditions prior to age 15	0.74	0.26
Number of types of chronic diseases	1.47	1.44
Binary variables	Frequency	Percentage (%)
Sex		
Male	917	48.85
Female	968	51.57
Marital status		
With spouse	1,245	66.33
No spouse	632	33.67
Household registration		
Urban	715	38.09
Rural	1,162	61.91
Pension		
Yes	1,558	83.00
No	319	17.00
Health insurance		
Yes	1,690	90.04
No	187	9.96
Smoking		
Yes	734	39.10
No	1,143	60.90
Drinking		
Yes	621	33.08
No	1,256	66.92
Quality of sleep		
Good	804	42.83
Bad	1,073	57.17
Toilet can be flushed		
Yes	817	43.53
No	1,060	56.47
Running water		
Can	1,338	71.28
Can not	539	28.72

## 3 Results

### 3.1 Socio-demographic, economic and lifestyle characteristics of participants

Based on the information in Table 2, it can be found that the age range of the respondents was 60 to 80 years, 48.48% of the older adult were male, 66.34% were accompanied by their spouses, 38.09% were urban older adult, 83% had a pension, 90.04% had a retirement pension, 60.92% were non-smokers, 66.91% were non-alcohol drinkers. 42.82% of the older adult have a good quality of sleep, 43.54% of the older adult have toilets that can be flushed, 71.26% of the older adult have access to running water, 74.18% of the older adult have a better endowment of health until the age of 15, and the average change of chronic diseases for the older adult of this age group is 1.4718 types.

### 3.2 Descriptive statistical results of health and physical activity at different ages

Based on the full-sample perspective, from the perspective of the mean values of the explanatory variables (I), regardless of the intensity of fitness activities, the overall health of the older adult who participate in physical fitness activities is greater than that of the older adult who do not, and it can be initially determined that participation in physical fitness activities improves the health of the older adult. The mean value of medical expenditures of the older adult who participated in sports and fitness activities was smaller than that of the older adult who did not participate in sports and fitness activities, which means that sports and fitness activities can reduce the medical burden of the older adult to a certain extent. In terms of the participation rate in physical fitness activities (II), the target of 50% for high-intensity fitness activities has not yet been reached in the 12th Five-Year Plan. However, the participation rate of medium-intensity and small-intensity fitness activities has exceeded 50%. In terms of exercise duration (III), older people who participate in physical fitness activities with higher intensity also exercise for a longer period of time. However, whether the level of health improves with the increase in the intensity and duration of exercise is yet to be further verified.

Mean health and mean healthcare expenditures were further analyzed under the physical fitness-age combination by grouping according to age. It was found that, first, the health status of older adults who participated in physical fitness activities was better than that of older adults who did not participate in physical fitness activities, regardless of the intensity of physical fitness activities, as the age group increased. Second, regardless of the intensity of physical fitness activities, the mean value of health care expenditures of older adults was reduced at different ages. However, with the increase of age, the degree of reduction of health care burden by participating in small-intensity fitness activities for the older adult showed an increasing trend. Therefore, the crowding-out effect of physical fitness on healthcare expenditures needs to be examined in terms of age heterogeneity.

In the table above, “Difference 1” represents the difference between the mean overall health of older persons who engaged in physical activity of a certain intensity and the mean overall health of older persons who did not engage in physical activity of that intensity; “Difference 2” represents the difference between the mean health care expenditures of older adults who engage in physical activity of a certain intensity and the mean health care expenditures of older adults who do not engage in physical activity of that intensity. Since the respondents may participate in physical fitness activities of various intensities, the total participation rate in physical fitness activities is more than 100%. The content of the table is derived from the author’s analysis.

### 3.3 The impact of physical fitness on the health of older adults

Table 3 presents the results of the impact of participating in physical fitness activities of varying intensities and durations on the health levels of the older adult. The dependent variable is the overall health level of the older adult. Regressions 1 to 3 report the impact effect of participating in physical fitness activities on health, and regressions 4 to 6 analyze the impact effect of the duration of physical fitness activities on health among participating older adult.

**Table 3:** Effects of physical fitness intensity and duration on health.

Variable	Physical fitness activity intensity			Duration of physical fitness activities		
	Regression 1	Regression 2	Regression 3	Regression 4	Regression 5	Regression 6
High-intensity fitness activity	0.0104^**^					
	(0.0052)					
Moderate-intensity fitness activity		0.0205^***^				
		(0.0048)				
Low-intensity fitness activity			0.0355^***^			
			(0.0086)			
Duration of high-intensity fitness activity				5.40*10^–4**^		
				(2.57*10^−4^)		
Duration of moderate-intensity fitness activity					5.11*10^–4**^	
					(2.25*10^−4^)	
Duration of low-intensity fitness activity						5.25*10^–5**^
						(2.05*10^−4^)
Age	−9.46*10^–4***^	−7.30*10^–4***^	−9.09*10^–4***^	−1.68*10^–3***^	−3.94*10^−4^	−6.27*10^–4***^
	(3.27*10^−4^)	(3.17*10^−4^)	(3.10*10^−4^)	(5.57*10^−4^)	(3.70*10^−4^)	(3.16*10^−4^)
Sex	0.0261^***^	0.0263^***^	0.0259^***^	0.0227^**^	0.0209^***^	0.0248^***^
	(0.0062)	(0.0062)	(0.0061)	(0.0093)	(0.0069)	(0.0061)
Marital status	0.0029	0.0026	0.0039	0.0042	−0.0019	0.0018
	(0.0052)	(0.0052)	(0.0052)	(0.0082)	(0.0061)	(0.0052)
Years of education	0.0035^***^	0.0034^***^	0.0033^***^	0.0016^*^	0.0021^***^	0.0033^***^
	(0.0006)	(0.0006)	(0.0006)	(0.0009)	(0.0007)	(0.0006)
Household registration	0.0029	0.0040	0.0022	0.0078	0.0062	0.0033
	(0.0053)	(0.0052)	(0.0052)	(0.0086)	(0.0060)	(0.0052)
Smoking	0.0045	0.0051	0.0046	0.0006	0.0033	0.0017
	(0.0058)	(0.0058)	(0.0058)	(0.0085)	(0.0064)	(0.0058)
Drinking	0.0130^**^	0.0119^**^	0.0134^***^	0.0209^***^	0.0124^**^	0.0131^**^
	(0.0051)	(0.0051)	(0.0050)	(0.0072)	(0.0056)	(0.0051)
Sleep quality	0.0178^***^	0.0175^***^	0.0178^***^	0.0145^**^	0.0176^***^	0.0164^***^
	(0.0044)	(0.0044)	(0.0044)	(0.0068)	(0.0051)	(0.0045)
Mensions	0.0138^*^	0.0136^*^	0.0134^*^	0.0052	0.0067	0.0149^*^
	(0.0073)	(0.0072)	(0.0073)	(0.0101)	(0.0081)	(0.0076)
Medical insurance	0.0104	0.0099	0.0093	−0.0079	0.0061	0.0055
	(0.0081)	(0.0081)	(0.0080)	(0.0121)	(0.0096)	(0.0080)
Logarithm of income	5.45*10^–4**^	4.78*10^–4**^	5.00*10^–4**^	1.59*10^–4***^	9.17*10^–4**^	2.76*10^–4**^
	(2.11*10^−4^)	(1.97*10^−4^)	(2.02*10^−4^)	(0.75*10^−4^)	(4.65*10^−4^)	(1.05*10^−4^)
Toilets flushed or not	0.0203^***^	0.0209^***^	0.0198^***^	0.0152^**^	0.0161^***^	0.0156^***^
	(0.0048)	(0.0048)	(0.0048)	(0.0073)	(0.0056)	(0.0050)
Availability of running water	0.0067	0.0061	0.0072	−0.0040	0.0032	0.0087
	(0.0055)	(0.0054)	(0.0054)	(0.0071)	(0.0062)	(0.0054)
Health status before age 15	0.0020	0.0014	0.0011	0.0080	0.0031	0.0004
	(0.0049)	(0.0049)	(0.0049)	(0.0077)	(0.0058)	(0.0051)
Chronic Diseases	−0.0147^***^	−0.0146^***^	−0.0146^***^	−0.0126^***^	−0.0137^***^	−0.0150^***^
	(0.0015)	(0.0015)	(0.0015)	(0.0023)	(0.0018)	(0.0016)
Constant	0.8440^***^	0.8190^***^	0.8130^***^	0.9440^***^	0.8460^***^	0.8360^***^
	(0.0259)	(0.0253)	(0.0260)	(0.0407)	(0.0306)	(0.0250)
R^2^	0.1720	0.1800	0.1830	0.1280	0.1400	0.1590
Sample Size	1877	1877	1877	730	1,215	1,671

* indicates *p*-value <0.1, ** indicates *p*-value <0.05, *** indicates *p*-value <0.01. The values in parentheses are robust standard deviations, same below. The content of the table is derived from the author’s analysis.

The results show that, after adding control variables, at the 5% significance level, firstly, the effect of participating in fitness activities of different intensities on the health level of the older adult is significant and the regression coefficients are positive relative to those who do not engage in physical fitness activities, indicating that physical fitness activities of different intensities are effective in improving the average health level of the older adult (see regressions 1–3 in Table 3). Secondly, for the older adult, it is not true that the greater the intensity of physical fitness activities the greater the improvement in health. From the regression coefficients, the degree of improvement in health from performing low-intensity fitness activities (regression coefficient of 0.0355, *p* < 0.01) is greater than that from moderate-intensity fitness activities (regression coefficient of 0.0205, *p* < 0.01), while moderate-intensity fitness activities improve health more than high-intensity fitness activities (regression coefficient of 0.0104, *p* < 0.1). As the age of the older adult increases, their mobility tends to decline, so the intensity of participation in physical activity decreases. This also explains, to some extent, the better health of older people who participate in physical fitness activities than those who do not, and suggests that older people are not suited to participating in high-intensity fitness activities.

Regression 4 to Regression 6 in Table 3 indicate the effect of exercise duration on health for different intensities of fitness activities. The results show that regardless of the type of fitness activity, the effect of improving health is greater for older adults who participate in physical fitness activities and the longer the duration of exercise. An increase of 1 h per week in participation in high intensity fitness activities increases the level of health by 0.000540 units (*p* < 0.05), while moderate intensity fitness activities and low intensity fitness activities increase the level of health by 0.000511 (*p* < 0.05) and 0.0000525 (*p* < 0.05) units, respectively.

### 3.4 The crowding out effect of physical activity on healthcare expenditures of the older adult

This paper includes variables affecting health in Model (2), as well as the dependent variable from Model (1), to control for the effects of health on healthcare expenditure. This is due to the statistical significance of overall health level and chronic diseases as important factors influencing healthcare expenditure. Since the overall health variable does not include aspects of chronic diseases, both are included as control variables in the model.

Regressions 1 to 3 in Table 4 report the marginal effects of participating in physical fitness activities on healthcare expenditure. After controlling for other variables affecting healthcare expenditure, the regression coefficients for physical fitness activities are significant and negative, indicating a significant inverse effect of participation in physical fitness activities on healthcare expenditure. In other words, the healthcare expenditure of older adult who engage in regular physical fitness activities is lower compared to those who do not participate. Specifically, participating in high-intensity fitness activities saves 0.0192 (*p* < 0.01) thousand RMB per month, medium-intensity activities save 0.0366 (*p* < 0.01) thousand RMB per month, and low-intensity activities save 0.0152 (*p* < 0.05) thousand RMB per month. Regressions 4 to 6 in Table 4 further measure the marginal effects of the duration of fitness activities of different intensities on healthcare expenditure. It is found that regardless of the intensity, increasing the duration of physical fitness activities significantly saves healthcare expenditure. For high-intensity activities, each additional hour per week saves 0.0014 (*p* < 0.05) thousand RMB per month, for medium-intensity activities, it saves 0.0021 (*p* < 0.05) thousand RMB per month, and for low-intensity activities, it saves 0.0016 (*p* < 0.05) thousand RMB per month. From a comprehensive analysis of these three aspects, there is sufficient reason to believe that physical fitness activities can effectively reduce the medical burden of China’s older adult. In other words, participating in physical fitness activities can lead to a “crowding-out effect” on healthcare expenditure, with the effect being more pronounced under medium-intensity fitness activities.

**Table 4:** Effects of physical fitness intensity and duration on healthcare expenses.

Variable	Regression 1	Regression 2	Regression 3	Regression 4	Regression 5	Regression 6
High-intensity fitness activity	−0.0192^***^					
	(0.0071)					
Moderate-intensity fitness activity		−0.0366^***^				
		(0.0142)				
Low-intensity fitness activity			−0.0152^**^			
			(0.0071)			
Duration of high-intensity fitness activity				−0.0014^**^		
				(0.0007)		
Duration of moderate-intensity fitness activity					−0.0021^**^	
					(0.0010)	
Duration of low-intensity fitness activity						−0.0016^**^
						(0.0009)
Overall health	−0.2300^**^	−0.2170^**^	−0.2390^**^	−0.1210^*^	−0.4090^**^	−0.2650^**^
	(0.1110)	(0.1060)	(0.1140)	(0.0648)	(0.2070)	(0.1220)
Age	−0.0076^***^	−0.0079^***^	−0.0073^***^	−0.0090^***^	−0.0109^***^	−0.0087^***^
	(0.0028)	(0.0026)	(0.0026)	(0.0029)	(0.0037)	(0.0029)
Sex	−0.0657	−0.0670	−0.0676	−0.0251	−0.0550	−0.0764
	(0.0564)	(0.0563)	(0.0562)	(0.0718)	(0.0625)	(0.0628)
Marital status	0.1390^***^	0.1380^***^	0.1390^***^	0.0225	0.0398	0.1350^***^
	(0.0485)	(0.0484)	(0.0486)	(0.0509)	(0.0621)	(0.0522)
Years of education	0.0160^**^	0.0163^***^	0.0162^***^	0.0061	0.0152^**^	0.0154^**^
	(0.0062)	(0.0063)	(0.0062)	(0.0064)	(0.0076)	(0.0067)
Household registration	0.0747^*^	0.0740^*^	0.0782^*^	0.0748	0.0754	0.0753^*^
	(0.0407)	(0.0404)	(0.0400)	(0.0502)	(0.0535)	(0.0444)
Smoking	−0.0258	−0.0272	−0.0263	−0.0330	−0.0328	−0.0255
	(0.0439)	(0.0443)	(0.0441)	(0.0507)	(0.0474)	(0.0478)
Drinking	−0.0791^*^	−0.0780^*^	−0.0796^*^	−0.0292	−0.0751	−0.0930^**^
	(0.0428)	(0.0429)	(0.0430)	(0.0529)	(0.0527)	(0.0468)
Sleep quality	0.0201	0.0206	0.0212	0.0069	0.0052	0.0339
	(0.0381)	(0.0378)	(0.0378)	(0.0409)	(0.0436)	(0.0413)
Mensions	0.0325	0.0332	0.0325	0.0344	0.1160^*^	0.0106
	(0.0610)	(0.0607)	(0.0611)	(0.0470)	(0.0638)	(0.0667)
Medical insurance	0.0356	0.0355	0.0335	0.1510^**^	0.0167	0.0640
	(0.0831)	(0.0837)	(0.0836)	(0.0699)	(0.1240)	(0.0947)
Logarithm of income	0.0095^**^	0.0096^**^	0.0095^**^	0.0100^**^	0.0125^**^	0.0120^**^
	(0.0048)	(0.0048)	(0.0048)	(0.0049)	(0.0063)	(0.0053)
Toilets flushed or not	−0.0211	−0.0221	−0.0203	0.0264	0.0245	−0.0249
	(0.0375)	(0.0376)	(0.0373)	(0.0411)	(0.0453)	(0.0405)
Availability of running water	−0.0763^*^	−0.0752^*^	−0.0755^*^	−0.0880^*^	−0.0412	−0.0681
	(0.0418)	(0.0416)	(0.0419)	(0.0476)	(0.0479)	(0.0454)
Health status before age 15	−0.0026	−0.0020	−0.0035	−0.0482	−0.0155	−0.0043
	(0.0467)	(0.0467)	(0.0469)	(0.0499)	(0.0623)	(0.0516)
Chronic diseases	0.0295^***^	0.0295^**^	0.0298^**^	0.0210^**^	0.0150^**^	0.0313^**^
	(0.0125)	(0.0135)	(0.0135)	(0.0110)	(0.0076)	(0.0152)
Constant	0.5820^**^	0.6040^**^	0.5430^**^	0.4310	0.9190^**^	0.6440^**^
	(0.2850)	(0.2710)	(0.2700)	(0.3040)	(0.4250)	(0.3020)
F-test	3.1800^***^	3.1100^***^	3.17000^***^	2.0200^***^	2.3000^***^	2.8700^***^
Sample size	1877	1877	1877	730	1,215	1,671

The content of the table is derived from the author’s analysis.

## 4 Discussion

### 4.1 The effect of physical activity on the health of older adults under different age groups

Table 1 indicates that age is a significant variable affecting health, with the overall health level of the older adult declining as age increases. To verify whether the impact of physical fitness activities changes with age, a more detailed investigation is conducted into the positive effects of physical fitness activities and their intensity and duration across different age groups. The regression analysis results are shown in Table 5. After controlling for related variables, the results of the impact of physical fitness activities on health indicate that high-intensity fitness activities significantly improve health only for middle-aged and younger seniors. For 60–65 year olds, high-intensity physical activity helps to improve their health by 0.0203 (*p* < 0.05) units. As the duration of high-intensity physical activity increases, an additional minute of exercise per week will improve the health of this group of older adults by an average of 3.20*10^−4^ (*p* < 0.01) units. For 66–70 older adults, high-intensity physical activity can help improve their health by 0.0034 (*p* < 0.05) units. However, this group of older adults should not be too involved in high-intensity physical activity, and High-Intensity Fitness Activity Duration does not have a significant effect on the improvement of health level at 5% level of significance. As for the 70–80 years olds, High-Intensity Fitness Activity Duration has no significant effect on the health level enhancement. By comparison, moderate-intensity physical activity and low-intensity fitness activity are appropriate for older adults of all ages at the 5% significance level. Cross-sectional comparisons show that 60–65 year olds are more suitable for higher intensity physical activity (impact coefficient of 0.0203, *p* < 0.05); 66–70 year olds are more suitable for moderate intensity physical activity (impact coefficient of 0.0053, *p* < 0.05); 71–75 year olds are more suitable for low-intensity fitness activity (impact coefficient of 0.0036, *p* < 0.05); and 76–80 year olds are more suitable for low-intensity fitness activity (impact coefficient of 0.0757, *p* < 0.05).

**Table 5:** Effects of physical activity on health at different age groups.

Variable	60–65 (years)			66–70 (years)		
Physical fitness activity intensity	Regression 1	Regression 2	Regression 3	Regression 1	Regression 2	Regression 3
High intensity	0.0203^**^			0.0034^***^		
Moderate intensity		0.0193^**^			0.0053^**^	
Low intensity			0.0172^**^			0.0260^**^
Exercise duration	Regression 4	Regression 5	Regression 6	Regression 4	Regression 5	Regression 6
High-intensity fitness activity duration	3.20*10^–4**^			8.91*10^–4*^		
Moderate-intensity fitness activity duration		3.24*10^–4**^			1.04*10^–3**^	
Low-intensity fitness activity duration			2.07*10^–4**^			1.11*10^–4**^
Variable	71-75(years)			76-80(years)		
Physical fitness activity intensity	Regression 1	Regression 2	Regression 3	Regression 1	Regression 2	Regression 3
High intensity	0.0111^*^			0.0145		
Moderate intensity		0.0204^*^			0.0398^**^	
Low intensity			0.0036^**^			0.0757^**^
Exercise duration	Regression 4	Regression 5	Regression 6	Regression 4	Regression 5	Regression 6
High-intensity fitness activity duration	9.06*10^–4*^			6.46*10^–4*^		
Moderate-intensity fitness activity duration		2.39*10^–4**^			3.35*10^–4**^	
Low-intensity fitness activity duration			3.59*10^–5**^			3.60*10^–5**^

The content of the table is derived from the author’s analysis.

In terms of the impact of the duration of physical fitness activities on health, the exercise duration of different intensities positively enhances health across all age groups. Consistent with the full sample analysis results, the longer the older adult participate in physical fitness activities, the greater the improvement in health levels. This suggests that during the implementation of the comprehensive fitness plan from 2011 to 2015, there was no over-exercising among the older adult in China. Overall, they were still within the positive benefit range of physical fitness activities. So increase the promotion of national fitness programs to raise awareness among the older adult about engaging in physical fitness activities. Government departments can use various media forms for promotion and publicity. Educating more older adult individuals about the benefits of exercise in enhancing personal health can motivate more older adult individuals to participate in national fitness programs.

### 4.2 The crowding out effect of physical activity on healthcare expenditures of the older adult across age groups

The results in Table 4 have confirmed that participation in physical fitness activities has a crowding-out effect on health care expenditure. So this paper further explores whether participation in physical fitness activities can directly contribute to a decrease in healthcare expenditure for the older adult, and whether increasing the intensity and duration of these activities can lead to a more significant reduction in healthcare costs. Focus on analyzing the heterogeneity of their age.

Building on the results presented in Table 6 reports the crowding-out effects of participation in physical fitness activities, the intensity of these activities, and their duration on healthcare expenditure for the older adult across different age groups. The findings for the crowding-out effect of physical fitness activities on healthcare expenditure for the older adult (Regressions 1 to 3) are similar to those of the full sample. Across all age groups, regular participation in physical fitness activities of varying intensities tends to reduce healthcare expenditure compared to those who do not participate. Specifically, the greatest crowding-out effect for persons aged 60–70 is seen with high-intensity activities (The regression coefficient is −0.0576, *p* < 0.05); for those aged 71–75, medium-intensity activities have the most significant effect (The regression coefficient is −0.0568, *p* < 0.01); and for the persons aged 76–80, low-intensity activities are most effective (The regression coefficient is −0.0475, *p* < 0.1). This suggests that older adult in different age brackets should engage in fitness activities of varying intensities to alleviate their healthcare expenditure burden to the greatest extent. Older adult who exercise regularly save an average of 0.008491 million USD per month in healthcare expenditures (27). Using the average exchange rate from the 2015 China Statistical Yearbook, 1 USD equals 6.2284 RMB, converting the above figure to 0.0529 thousand RMB per month. Using this as a benchmark, it is evident that the crowding-out effect of fitness activities on healthcare expenditure exists for Chinese older adult, but the magnitude is smaller than the corresponding effect for American older adult. So encourage proactive health management by reducing healthcare expenditure through participation in physical fitness activities. Compared to the use of medical services, which is often passive, short-term beneficial, and costly, physical activity or exercise is proactive, offers long-term benefits, and is less expensive. To address the healthcare expenditure burden brought by aging, relevant government departments should shift their policy-making focus from treatment to prevention.

**Table 6:** Effects of physical activity on healthcare expenses at different ages.

Variable	60–65 (years)			66–70 (years)		
Physical fitness activity intensity	Regression 1	Regression 2	Regression 3	Regression 1	Regression 2	Regression 3
High intensity	−0.0576^**^			−0.0138^*^		
Moderate intensity		−0.0345^**^			−0.0114^**^	
Low intensity			−0.0099^**^			−0.0118^**^
Control variables	Yes	Yes	Yes	Yes	Yes	Yes
Exercise duration	Regression 4	Regression 5	Regression 6	Regression 4	Regression 5	Regression 6
High-intensity fitness activity duration	−0.0079^**^			−0.0048^**^		
Moderate-intensity fitness activity duration		−0.0025^**^			−0.0059^*^	
Low-intensity fitness activity duration			−9.39*10^–4*^			−0.0051^**^
Control variables	Yes	Yes	Yes	Yes	Yes	Yes
Variable	71-75(years)			76-80(years)		
Physical fitness activity intensity	Regression 1	Regression 2	Regression 3	Regression 1	Regression 2	Regression 3
High intensity	−0.0166^**^			−0.0040^*^		
Moderate intensity		−0.0568^*^			−0.0373^**^	
Low intensity			−0.0124^**^			−0.0475^*^
Control variables	Yes	Yes	Yes	Yes	Yes	Yes
Exercise duration	Regression 4	Regression 5	Regression 6	Regression 4	Regression 5	Regression 6
High-intensity fitness activity duration	−9.95*10^–4*^			−0.0002^*^		
Moderate-intensity fitness activity duration		−0.0034^**^			−0.0006^*^	
Low-intensity fitness activity duration			−0.0010^**^			−0.0023^**^
Control variables	Yes	Yes	Yes	Yes	Yes	Yes

The content of the table is derived from the author’s analysis.

### 4.3 The crowding out effect of duration of physical fitness activities on healthcare expenditure for the older adult

Regarding the crowding-out effect of the duration of physical fitness activities on healthcare expenditure for the older adult (Regressions 4 to 6), the maximum values indicate that for each additional hour per week of high-intensity fitness activities, healthcare expenditure for individuals aged 60–65 can be reduced by 0.0079 (*p* < 0.05) thousand RMB per month. For medium-intensity activities, each additional hour per week can save 0.0059 (*p* < 0.1) thousand RMB per month for those aged 66–70 and 0.0034 (*p* < 0.05) thousand RMB per month for those aged 71–75. For low-intensity activities, each additional hour per week can save 0.0023 (*p* < 0.05) thousand RMB per month for individuals aged 76–80. Overall, as age increases, the crowding-out effect of high-intensity fitness activities on healthcare expenditure tends to decrease, while the effects of low and medium-intensity activities increase. This indicates that different age groups of older adult are suited for different intensities of physical fitness activities. With increasing age, the intensity of physical fitness activities should be reduced. Specifically, high-intensity fitness activities are more suitable for younger persons aged 60–70, medium-intensity activities for those aged 66–75, and low-intensity activities for the older adult aged 76–80. So government departments can design a set of recommended exercise intensity guidelines for older adult across different age groups. Relevant departments in China should also compile a “Physical Activity Guide for the older adult,” tailored to the unique physical conditions of the Chinese older adult. This guide would provide recommended physical fitness activity plans for older adult of different ages, offering suggestions for exercise intensity, duration, frequency, and types of activities.

## 5 Limitations and future research

Despite its contribution, this study has some limitations. First, this paper examines the base effects of different physical activities on health and healthcare expenditures only for Chinese 60–80 year olds. No comparisons were made to developed countries around the world. In the future, the extent of the “crowding out effect” can be compared between developed countries and China. Second, this paper uses 2015 data from the CHARLS database. Data from other years do not provide as comprehensive information on physical education activities as the 2015 data. Although it is not the most recent data, the sample size is large enough to achieve the purpose of this paper, which is to verify the “crowding out effect.” Third, due to the limitation of data, this paper only studies three kinds of intensity of physical activities, and Chinese older adult people’s physical activities are more diversified, such as: martial arts, square dance, etc. In the future, similar surveys can be carried out to further study the effects of different forms of physical activities on health.

## 6 Conclusion

Physical fitness activities significantly improve the health level of the older adult. Compared to the health level of older adult who do not participate in physical fitness activities, those who actively engage in such activities have a higher average health level. However, as the intensity of physical fitness activities increases, the degree of improvement in overall health decreases. Participation in physical fitness activities leads to a “crowding-out effect” on the healthcare expenditure of the older adult. Compared to the healthcare expenditure of older adult who do not participate in physical fitness activities, those who do participate see a significant reduction in healthcare costs. The longer the duration of the activity, the greater the reduction in healthcare expenditure. The effectiveness of different types of physical fitness activities varies significantly among different age groups of the older adult. Older adult in different age groups should choose physical activities of varying intensities based on their circumstances. Young and middle-aged older adult is suitable for high-intensity fitness activities, middle-aged and older adult for medium-intensity activities, and older adult for low-intensity activities.

## Data Availability

The original contributions presented in the study are included in the article/supplementary material, further inquiries can be directed to the corresponding author.
